# Structural and biomechanical alterations in rabbit thoracic aortas are associated with the progression of atherosclerosis

**DOI:** 10.1186/1476-511X-10-125

**Published:** 2011-07-26

**Authors:** Ioanna Koniari, Dimosthenis Mavrilas, Helen Papadaki, Menelaos Karanikolas, Martha Mandellou, Apostolos Papalois, Efstratios Koletsis, Dimitrios Dougenis, Efstratios Apostolakis

**Affiliations:** 1 Cardiothoracic Surgery Department, University Hospital of Patras, 26504 Patras, Greece; 2 Laboratory of Biomechanics and Biomedical Engineering, Department of Mechanical Engineering and Aeronautics, University of Patras, 26504, Patras, Greece; 3 Anatomy Department, University of Patras, 26504 Patras, Greece; 4 Department of Anaesthesiology and Critical Care Medicine, University Hospital of Patras, 26504 Patras, Greece; 5 Biochemistry Department, University Hospital of Patras, 26504 Patras, Greece; 6 Experimental Research Center, ELPEN Pharmaceuticals, Marathonos 95 str, 19009 Athens, Greece

## Abstract

**Background:**

Atherosclerosis is a diffuse and highly variable disease of arteries that alters the mechanical properties of the vessel wall through highly variable changes in its cellular composition and histological structure. We have analyzed the effects of acute atherosclerotic changes on the mechanical properties of the descending thoracic aorta of rabbits fed a 4% cholesterol diet.

**Methods:**

Two groups of eight male New Zealand White rabbits were randomly selected and fed for 8 weeks either an atherogenic diet (4% cholesterol plus regular rabbit chow), or regular chow. Animals were sacrificed after 8 weeks, and the descending thoracic aortas were excised for pressure-diameter tests and histological evaluation to examine the relationship between aortic elastic properties and atherosclerotic lesions.

**Results:**

All rabbits fed the high-cholesterol diet developed either intermediate or advanced atherosclerotic lesions, particularly American Heart Association-type III and IV, which were fatty and contained abundant lipid-filled foam cells (RAM 11-positive cells) and fewer SMCs with solid-like actin staining (HHF-35-positive cells). In contrast, rabbits fed a normal diet had no visible atherosclerotic changes. The atherosclerotic lesions correlated with a statistically significant decrease in mean vessel wall stiffness in the cholesterol-fed rabbits (51.52 ± 8.76 kPa) compared to the control animals (68.98 ± 11.98 kPa), especially in rabbits with more progressive disease.

**Conclusions:**

Notably, stiffness appears to decrease with the progression of atherosclerosis after the 8-week period.

## Background

Atherosclerosis is a progressive inflammatory process involving complex interactions between elevated plasma cholesterol and low-density lipoprotein (LDL) levels, arterial endothelial and smooth muscle cells (ECs and SMCs, respectively), migratory inflammatory cells (monocytes, T lymphocytes, platelets), and the pro-inflammatory cytokines that they release [1,2]. According to the response to injury hypothesis, atherosclerosis is triggered by factors, such as hyperlipidemia or mechanical injury, that stimulate endothelial dysfunction, the migration and proliferation of monocytes and SMCs into subendothelial spaces, followed by neointimal hyperplasia, plaque fibrosis and calcification [3,4]. Atherosclerotic lesions are highly variable in morphology at different stages of disease progression and are structurally heterogeneous, anisotropic, and incompressible [5-7]. These pathophysiologic changes alter the morphology, material properties, and mechanical behavior of the arterial wall [8-10], which becomes non-linearly viscoelastic and subject to large strain deformations that induce additional rheological abnormalities [7].

Many in vitro and in vivo studies have demonstrated changes in the biomechanical properties of the aortic wall due to pathological conditions such as hypertension and atherosclerosis [11]. High resolution echo tracking technology [12-14] has shown reduced elasticity (stiffening) of arterial walls in vivo, particularly at sites of plaque formation. Mechanical testing has been used in vitro to study strips of arterial wall under uniaxial tension [15,16] or whole arterial segments dilated by pressurized fluid [7,8]. Typically these studies used animals with advanced disease and the aortic wall was found to be stiffer than normal, possibly due to the fibrous change and calcification found in the advanced lesions.

Here we used a quasi-physiological method to measure elasticity of pressurized segments of the thoracic aorta from New Zealand rabbits fed a high cholesterol diet. Using this method, we identified decreases in arterial wall stiffness associated with the progression and histological features of atherosclerotic lesions.

## Methods

### Animal model and specimens

The study was conducted in accordance to the Institutional "Guide for the Care and Use of Laboratory Animals" and was approved by the Institutional Animal Care and Use Committee of the West Greece Prefecture and the University of Patras. All experiments were performed in the Animal House of the Medical School of Patras University. Sixteen male New Zealand White rabbits, weighing 3-3.5 kg, were fed regular rabbit chow for one week, randomly divided into two treatment groups of eight animals and then fed either an atherogenic diet (Group A, 4% cholesterol plus regular rabbit chow, without additional atherogenic components; ELPEN pharmaceutical, Athens, Greece) or regular chow (Group B) for 8 weeks. Rabbits were housed individually at 20 ± 3°C with a 12-h:12-h light/dark cycle and with free access to water. Feeding was restricted to 120 g/day. Blood samples were collected every 4 weeks. The general appearance of the rabbits was observed daily. Body weights were measured every 4 weeks.

Rabbits were anesthetized with ketamine (50 mg/kg) plus xylazine (10 mg/kg) intramuscularly, and sacrificed by intravenous injection of a saturated KCl solution. The descending thoracic aorta was exposed, and a 50 mm segment was labeled in situ with two reference dots, cut at these points and excised. The marks guided us to prestrain the vessel segment *in vitro *so as to retain the above mentioned physiological length in situ [8]. External fatty layers were trimmed, and lateral branches were ligated. The specimens were stored in normal saline 0.9% at 4°C prior to mechanical testing. All mechanical tests were performed immediately after animal sacrifice.

### Experimental system and mechanical tests

**Figure 1:**
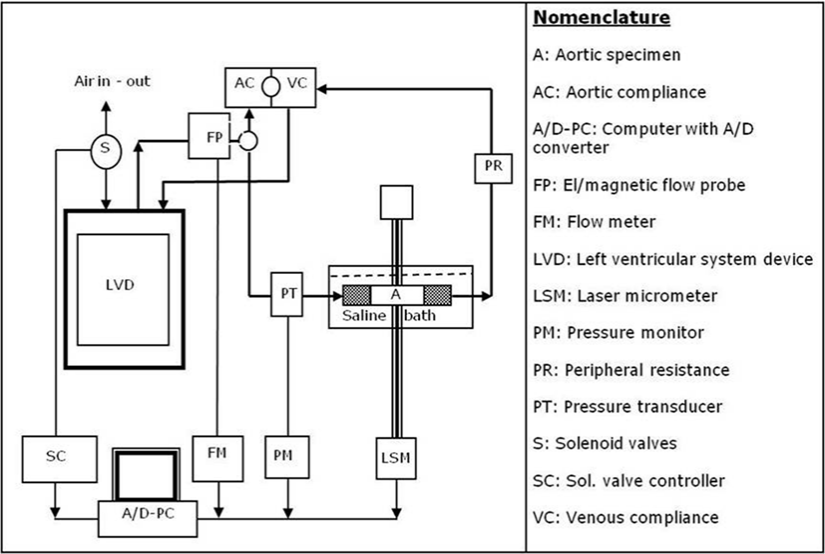
**A schematic diagram of the quasi-physiological closed loop flow test system**.

**Figure 2:**
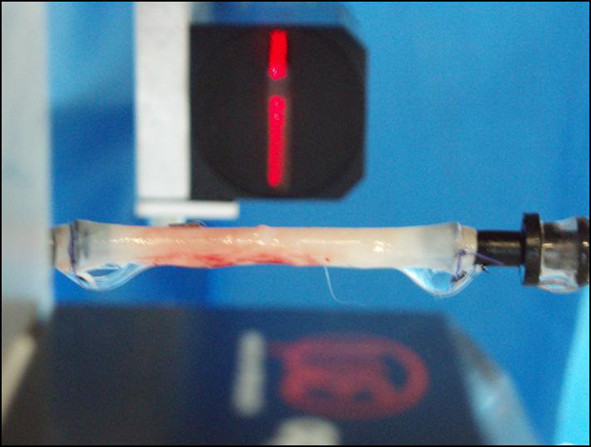
**Aortic specimen in place, out of saline bath before testing**. The red light beam of the laser micrometer and the corresponding shadow, depicting vessel's external diameter, are demonstrated.

The cut ends of each arterial segment were carefully secured in plastic hollow nozzles of proper size and connected to the "arterial branch" of a custom designed, closed loop mock circulatory system consisting of a left ventricular device (LVD) connected to hydraulic circuit of transparent, rigid plastic tubes. A diagram of the apparatus is presented in figure 1. The LVD was composed of a pneumatic silicon rubber sack enclosed in a tightened box, with mechanical disc valves at the inlet and outlet to produce unidirectional fluid flow in the closed circuit. A custom electronic controller (SC) stimulated two solenoid valves (S) working sequentially in opposite on-off mode. A pressurized air line connected to the mid-space between the rubber sack and the rigid box of the LVD was used to produce alternating compression and expansion of the LVD at time-periods 1/3 and 2/3 of the cycle, respectively. Windkessel elements (air-liquid interface in closed vessels connected with the hydraulic line) at the "proximal" (AC) and "distal" (VC) ends of the aortic segment, and flow resistance elements (PR) produced the corresponding vascular compliance and peripheral resistance. Arterial pressure was monitored invasively at the proximal end of the aortic segment using an electronic manometer (PM, CYQ 103 bridge transducer interface, Cybersience Inc. USA) and a Deltran DP-100 pressure transducer (PT, Utah Medical Products USA). External vessel diameter was measured using a 30-mm range laser micrometer (LSM, micro-epsilon GMBH, optocontrol type ODC 1201-30; Figure 2). Arterial flow was monitored using a cannulated medical electromagnetic flow meter (FM501) with a 300 A 1/2" flow probe (Karolina Medical Electronics, USA). All electronic analog voltage signals (pressure, diameter, and flow) were recorded electronically via an A/D converter card (DI 700, Data Q, USA). Raw data (in volts) were converted into mechanical units using appropriate calibration factors and used for mechanical analysis.

**Figure 3:**
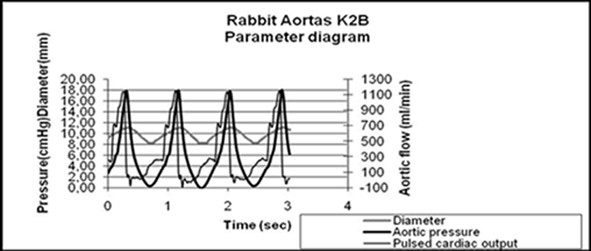
**Representative flow, pressure, and diameter traces with time for four consecutive cycles**.

For mechanical testing, aortic segments were positioned into the hydraulic system and the whole circuit was filled with normal saline at room temperature. The aortic specimens, after physiological prestraining, were subjected to pulsatile flow of 400 ml/min (mean arterial flow), at 60 cycles/min, with peak systolic/diastolic pressure values of 180/0^+ ^mmHg respectively. The diastolic pressure was non physiological low (approximately 0 mmHg), to maximize the pressure-dilation range of mechanical testing. Representative flow, pressure, and diameter traces for four consecutive cycles are shown in Figure 3.

### Biomechanical analysis

Different expressions of arterial wall elasticity have been introduced, using complicated constitutive equations of axial and peripheral stress and strain, so as to describe mechanical properties of arterial wall [17]. Pressure-strain elastic modulus (Peterson's modulus; E_P_) is an expression of arterial wall stiffness, according the following relationship:

where D_0 _is the outer vessel diameter at a certain P and ΔP and ΔD are the changes in pressure and diameter [18]. For small increments of P and D, E_P _is represented by the slope of the tangential line to the loading portion of the P- (D/D_0_) diagrams at a certain (P, D/D_0_) point of the pressure-dilatation curve.

In present study, experimental data of pressure and external diameter were further analyzed to calculate a modified arterial stiffness E_Pm_. External diameter (D) measurements, ranged from the minimum diameter D_min _at minimum internal pressure (0^+ ^mmHg) to that at maximum applied pressure, were divided by a reference pressure, (D_S_), so as to compute normalized radial arterial dilatation (RAD) as follows:

where D is the external diameter at minimum or maximum internal pressures, and D_S _is the external diameter at a reference pressure of 100 mmHg [5]. Hence, E_Pm _at each pressure was computed as:

### Biochemical assays

Total serum cholesterol (TC), triglyceride (TG), and high density lipoprotein (HDL) levels were used to measure hyperlipidemia, and renal and liver function were monitored using urine, creatinine and serum hepatic enzymes (SGPT and g-GT) levels, respectively.

### Histological analysis

After mechanical testing, vessels were fixed by immersion in neutrally buffered 10% formalin, followed by dehydration and embedding in paraffin wax using standard procedures. Four-micrometer sections were obtained from each vessel at 5-mm intervals and stained with hematoxylin and eosin for histopathologic analysis. Masson's trichrome aniline blue and Weigert Van Gieson's elastic stains (Histoline Laboratories, Italy) were used to assay collagen components and the thickness of the intima, respectively.

### Immunohistochemistry

Consecutive 4-μm-thick sections from each aortic specimen were collected on Superfrost plus glass slides, deparaffinized, and rehydrated in graded alcohols. Endogenous peroxidase activity was blocked by treatment with 3% hydrogen peroxide for 15 min, followed by incubation with protein blocking solution to eliminate nonspecific binding. Immunohistochemistry was performed using monoclonal antibodies to detect macrophages (RAM 11, 1:200 dilution; Dako Corp, CA, USA), and α-actin in SMCs (HHF-35, 1:100 dilution; DAKO A/S). The Envision Plus Detection System kit (DakoCytomation, USA) and 3, 3'-diaminobenzidine (DAB) were used to visualize antibody binding, according to manufacturer's instructions. Sections were counterstained with Harris' hematoxylin, dehydrated, and mounted permanently. For each antibody, all tissues from the different study animals were immunostained concurrently. Negative controls were performed in all cases by omitting the primary antibodies.

### Statistical analysis

SPSS for Windows (release 17. 0. 0 SPSS Inc, Chicago IL, USA) was used for continuous data analysis. All data are expressed as the mean ± standard deviation (SD). Statistical significance was determined using a two-tailed Student t test. A p value < 0.05 denotes statistical significance.

## Results

### Atherogenic diet resulted in hypercholesterolemia

In rabbits of group A fed the atherogenic diet, increases in mean TC, TG, and HDL levels (mg/dl) from 4 to 8 weeks were as follows: TC increased from 4121 ± 415 to 4423 ± 493.40, TG from 381 ± 54.60 to 502 ± 96.24, and HDL from 383 ± 40.50 to 412 ± 15.40 (Table 1). In control animals (group B), TC and TG increased slightly after 8 weeks but remained within the normal range, revealing a statistical significant difference (p < 0.001) between the two groups (Table 1). At the end of the 8-week period, there was no statistically significant change in body weight between the hypercholesterolemic and control rabbits (3600 ± 92.58 g and 3706 ± 152.20 g, respectively), and the animals on the atherogenic diet had normal renal and liver function, confirming that the 4% cholesterol diet was not overtly toxic.

**Table 1:** Blood assays of cholesterol-fed and normal rabbits

	Group A		Group B	
	
Blood assays	4 weeks	8 weeks	4 weeks	8 weeks
**TC**	4121 ± 415	4423 ± 493.40	35 ± 7.25	55.5 ± 11.82

**TG**	381 ± 54.60	502 ± 96.24	38 ± 2	43.8 ± 9.66

**HDL**	383 ± 40.50	412 ± 15.40	21.8 ± 3.60	34.5 ± 2.88

**SGPT**	46.5 ± 5.53	53.75 ± 5.57	32.5 ± 8	39.7 ± 6

**g-GT**	6.25 ± 2.05	7.25 ± 1.58	4.25 ± 0.89	5.25 ± 0.89

**Urine**	38.75 ± 2.87	39.25 ± 2.43	42.25 ± 2.87	44.25 ± 7.10

**Creatinine**	0.79 ± 0.11	0.89 ± 0.13	0.78 ± 0.46	0.93 ± 0.18

### Hypercholesterolemia was associated with intermediate or advanced atheroma formation

**Figure 4:**
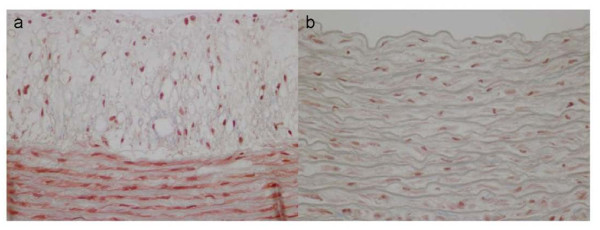
**Masson's trichrome staining**. a. Slight connective tissue increment in aortic atherosclerotic lesion of group A b. No histopathological changes in control aortas.

**Figure 5:**
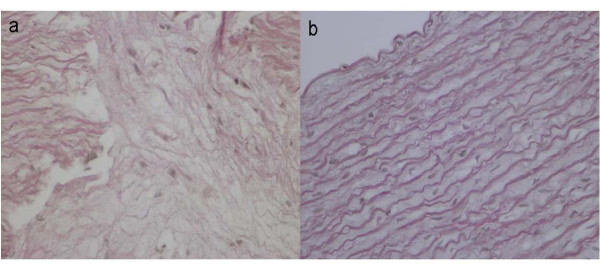
**Van Gieson staining**. a. Focal fragmentation and disorientation of elastic fibres, close to atheromatous lesion in Group A b. Normal elastic fibres of control aortas.

Hematoxylin and eosin-stained sections of aorta were examined for signs of atheroma. Whereas animals on the normal diet had no visible atheroma, the atherogenic diet induced a statistically significant incidence of atherosclerotic lesions in all group A animals (p < 0.001). Lesions were classified according to the guidelines of the American Heart Association [19] as intermediate (type III, n = 4 animals) or advanced (mostly type IV; n = 4 animals). Type III lesions consisted of intimal hyperplasia, foam cells, SMCs with visible lipid inclusions, and extracellular deposits of lipid. Type IV lesions consisted of nuclear lipid droplets, lack of any marked fibrous tissue, extracellular matrix rich in proteoglycans, and abundant infiltrating macrophages, lymphocytes, and foam cells [19]. Notably, type IV is the first lesion considered advanced in this classification because of the severe intimal disorganization caused by the lipid core. The characteristic core appears to develop from an increase and the consequent confluence of the small isolated pools of extracellular lipid that characterize type III lesions. The increase in lipid is believed to result from continued insudation from the plasma [19]. Masson and Van Gieson staining revealed a slight deposition of collagen tissue in tunica media and intima, as well as disruption of elastic fibers in internal elastic lamina of atherosclerotic aortas, whereas no such changes were observed in aortas from group B (Figures 4 and 5). The severity of the aortic atherosclerotic lesions was positively correlated with the high total cholesterol serum level.

**Figure 6:**
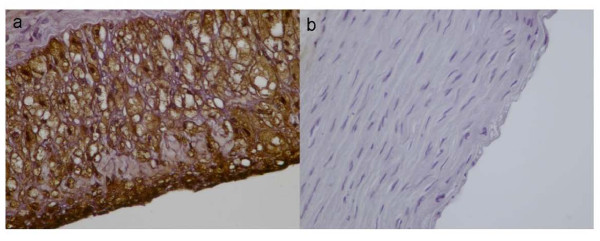
**Ram -11 staining**. a. RAM-11 positive foam cells in atherosclerotic lesions of group A b. lack of foam cells in normal aortic wall.

Immunohistochemical staining was graded on a scale of 0 to 3 based on the percentage of immunopositive cells as follows: 0, <10% positive cells; 1 (mildly positive), 10-35% positive cells; 2 (moderately positive), 35-70% positive cells; and 3 (strongly positive), >70% positive cells. The high cholesterol diet was associated with a significant increase in lipid deposition and foam cell formation, indicated by the increase in RAM-11 immunoreactivity in animals of group A (Table 2). The aortas from all animals of group A were strongly positive for RAM-11 staining, whereas no staining was observed in aortas of control animals (Figure 6). We also observed that the number of RAM-11 positive cells correlated with the severity of the atherosclerotic lesions.

**Table 2:** Immunostaining scores for RAM-11 and HHF-35 in hyperlipidemic and control animals

	Group A		Group B	
	
IHC score	RAM-11	HHF-35	RAM-11	HHF-35
Negative	0	0	8 (100%)	8(100%)

Mildly positive	0	4(50%)	0	0

Moderately positive	0	4(50%)	0	0

Strongly positive	8 (100%)	0	0	0

Total number	8 (100%)	8(100%)	8(100%)	8 (100%)

**Figure 7:**
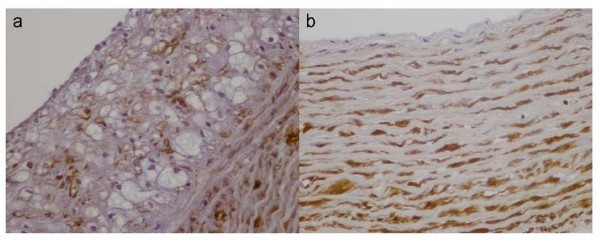
**HHF-35 staining**. a. Positive for a- actin SMCs are observed between foam cells in the atherosclerotic lesions of group A b. HHF-35 positive SMCs are normally noticed in the media of control aortas.

HHF-35 immunostaining for α-actin revealed that the aortic atherosclerotic lesions of all hypercholesterolemic animals contained either mild (n = 4 animals) or moderate (n = 4) numbers of SMCs, compared with 0% in control animals (Figure 7). Moreover, the percentage of SMCs correlated with the severity of atherosclerosis.

### Atherosclerosis reduced the wall stiffness of the aorta

**Figure 8:**
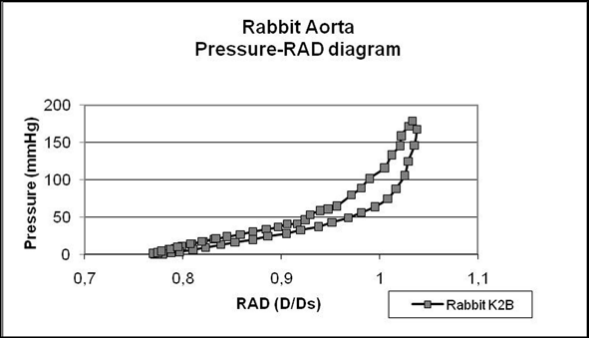
**Representative diagram of Pressure/RAD from one animal (K2B)**. Diameter D is normalized (D/Ds) with respect to the external diameter Ds at pressure level 100 mmHg.

The aorta segments were subjected to alternating cycles of physiological systole and diastole, and the changes in external vessel wall diameter were measured. During each pressure cycle we observed a >30% increase in external vessel diameter at peak pressure (Dmax) in comparison with the diameter at minimum pressure (Dmin). Plotting pressure vs. RAD demonstrated a nonlinear relationship between 0-200 mmHg (Figure 8), similar to stress-strain curves of other soft tissues. During each pressure cycle, the pressure loading curve followed a different pathway compared to pressure unloading. This loop relationship indicates energy dissipation during each pressure cycle and demonstrates the viscoelastic characteristics of the arterial wall [20,21]. In contrast to the nonlinear curve observed over in the entire pressure range, the relationship between pressure and RAD was close to linear at physiological pressures 50-150 mmHg (correlation coefficient R2 of the linear fitting curve equation was computed with values > 0.8). Rather than calculate E_Pm _values at particular pressures [18], we determined average E_Pm _throughout the linear range. Diameter measurements were performed for 50 sequential pressure cycles at the proximal, center and distal portions of each vessel. Sets of at least five consecutive stable cycles were identified, and the slope value in each position was computed from a single pressure cycle from each set. The mean stiffness of the local slope values at all three locations were derived for each specimen.

**Figure 9:**
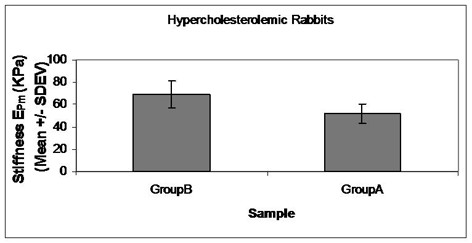
**Mean stiffness E_**Pm **_over the pressure range of 50-150 mmHg (mean ± SD, n = 8, p < 0.05)**.

These results demonstrated that the atherogenic diet resulted in a significant decrease of mean stiffness (51.52 ± 8.76 kPa; p = 0.005) compared to control animals (68.98 ± 11.98 kPa; Figure 9). Comparing the mechanical properties and histological changes, we found that the decrease in wall stiffness was negatively correlated with the severity of the atherosclerosis after the 8-week feeding period, as atherosclerotic lesions are mainly fatty, composed of foam cells and high extracellular lipid accumulation, without any marked fibrous tissue formation, as determined by Masson staining, that might increase aortic stiffness (type III and IV lesions).

## Discussion

We used quasi-physiologic conditions and a closed loop flow system to measure the pressure-diameter relationship in segments of thoracic aortas from a New Zealand rabbit model of induced atherosclerosis. After 8 weeks of a 4% high-cholesterol diet without additional atherogenic components, we found that the aortic elastic modulus decreased as the atherosclerosis became more advanced, specifically with the appearance of intermediate (type III) and early advanced (type IV) atherosclerotic lesions. Histologically, these lesions were fatty, composed mainly of foam cells filled with lipid (RAM 11-positive cells), and with a lesser number of SMCs rich in solid-like actin (HHF-35 positive cells); while fibrous change was notably absent. Especially, in type IV atherosclerotic lesions, the usual intimal smooth muscle cells and the intercellular matrix of the deep intima are dispersed and replaced by accumulated particles of extracellular lipid. Between the lipid core and the endothelial surface, the intima contains macrophages and smooth muscle cells with and without lipid droplet inclusions. Much of the tissue between the core and the surface endothelium corresponds to the proteoglycan-rich layer of the intima, although infiltrated with the cells as macrophages, foam cells, and lymphocytes are more densely concentrated in the lesion periphery [19]. Taken together, these factors could account for the observed decreases in stiffness. The potential clinical significance of decreased stiffness can be defined considering the histological basis of atherosclerotic lesions. Taking into account, that the region between the lipid core and the lesion surface contains proteoglycans and macrophage foam cells and only isolated smooth muscle cells and minimal collagen (potential mechanism of decreased stiffness), it may be susceptible to formation of fissures, intramural hematomas, aortic dilatation or even dissection. Thus, the periphery of advanced lesions, particularly type IV, may be vulnerable to rupture because macrophages are generally abundant in this location.

Our results are in accordance with previous studies of the mechanical properties of arterial segments with atherosclerosis. Firstly, Newman et al [22] reported that structural stiffness and wall elasticity of abdominal aorta was decreased during the earliest stages of atherosclerosis in cockerels fed an atherogenic diet. The decreased stiffness accompanying early atherosclerosis was attributed to the weakening of medial interlamellar elastic tissue-collagen network, probably due to lipid infiltration, whereas in advanced disease with calcification and fibrosis, aortic structural stiffness was higher than the control aortas. Nichol [23] reported that atherosclerosis reduced aortic stiffness at pressures below 70 mmHg, due to the destruction of elastic tissues. In a recent study designed to represent early and intermediate atherosclerosis, Hayashi and Imai [15] demonstrated that the force-deformation characteristics of atherosclerotic specimens of denuded thoracic rabbit aorta were less stiff than controls. Moreover, Hamilton et al [7] used intravascular ultrasound (IVUS) imaging, 3D reconstruction, and finite element analysis (FEA) to examine alterations in denuded femoral artery wall of Yucatan pigs fed a high cholesterol diet. The elastic modulus of the non-denuded femoral arteries decreased significantly on the high cholesterol diet, in agreement with our results. The decrease in elastic modulus with early/intermediate (fatty and fibrofatty lesions) atheroma was reversed and then increased as the lesions became fibrotic. Similarly, Vonesh et al [24] used IVUS image data with FEA to perform 3D reconstruction of human atherosclerotic segments of iliac and femoral arteries respectively, and found that the elastic modulus of non-atherosclerotic tissue regions was greater than early lipidous atherosclerotic regions for transmural pressure load at normal and hypertensive pressures (80-160 mmHg). De Korte et al [25] also used IVUS elastography of diseased human femoral and coronary arteries at 80 and 100 mmHg, and then compared the differences in strain between normal and diseased tissue. They reported that the pressure-strain modulus of fibrous tissue was double the modulus of fatty tissue, indicating the vulnerability of fatty lesions with only a thin fibrous cap. A recent study [26] used IVUS elastography to analyze denuded iliac and femoral arteries of Yacatan pigs on an atherogenic diet for 9-10 months. The mean strain value of arteries with early fat lesions (0.46) was greater that non diseased arteries (0.21) or diseased arteries with fibrous lesions (0.24), in agreement with our findings. Finally, Matsumoto et al [27] used pipette aspiration to measure the local elastic modulus of rabbit thoracic aortas fed a 1% cholesterol diet for 8-28 weeks. Similar to our results, the local elastic modulus of vessels with early lesions after 8 weeks of the atherogenic diet were significantly lower than normal tissue, although the modulus significantly increased after 24-28 weeks. The local elastic modulus appeared to decrease concurrently with the formation of early atherosclerotic lesions (intimal hyperplasia filled with foam cells), and increased gradually, coincident with the appearance of SMCs and calcification.

Our results conflict with a large number of studies that have used a variety of methods to examine the elastic properties of atherosclerotic artery walls in monkeys on high cholesterol diets for between 18-38 months. However, the contradiction with our findings is reasonable, as the duration of high cholesterol diet for atherosclerosis induction was much longer. Contrary to our findings, these studies found that arterial wall stiffness increases and decreases with the progression or regression of the atherosclerosis [28-30], respectively. It is possible that the increased stiffness after years of high cholesterol diet was due to the presence of highly fibrotic and calcified atherosclerotic lesions that would have an elastic modulus comparable to that of bone [27]. Others have found similar changes in short duration studies of rabbits or rats [31-34]. Interestingly, Hayashi et al. [8] found that arterial stiffness in rabbits fed a high cholesterol diet for 4-32 weeks did not increase unless there was also considerable calcification and wall thickening, even if the atherosclerosis was highly advanced. Finally, Richter and Mittermayer [35] observed that the modulus of volume elasticity of autopsied human aortas was higher in more advanced stages of atherosclerosis. It is possible that the tangential strips of aorta used in some studies [31,32] may behave differently in tests of elasticity than intact cylindrical arterial segments, such as we have used here, since it is difficult to set up isolated strips of tissue in a mechanical state that is comparable to the physiological loading conditions in vivo.

These conflicting results might also be attributed to different definitions or measurement of stiffness, testing methodologies, or stage of atherosclerosis examined. Although stiffness is generally described as a reflection of the arterial wall tissue rigidity, there are significant differences in how it is defined. While wall elasticity involves direct or indirect measurement of wall thickness, expressing the material properties of wall; stiffness, expressed as more or less similar to Peterson modulus E_P_, treats the vessel as a whole geometric-material structure. Thus, the different in vitro methods of physiological loading such as closed- or open-ended tube, with or without restriction of axial movement may also contribute to variability in the mechanical characteristics of the arterial wall. Determination of wall stiffness instead of wall elasticity in conjunction with the fixed ends of the aortic specimens during mechanical testing, that constrict the axial deformation occurred in vivo, could be regarded as limitations of our in vitro model.

## Conclusions

In conclusion, we found that the material properties of the aortic wall altered with the degree of progression of atherosclerotic disease and the corresponding changes in arterial wall morphology. The presence of early atherosclerotic lesions, characterized by abundant fatty cells, few SMCs and the partial loss of integrity in the elastin-collagen network, led to the histological reorganization of the aortic wall and the initial decrease of wall stiffness. Thus, it will be interesting to study the potential impact of agents such as statins on the structural and biomechanical properties of atherosclerotic aortic wall, and their potential contribution to plaque stabilization or even regression.

## List of abbreviations

D: external diameter; D_min: _minimum diameter; D_S_: external diameter at a reference pressure; ECs: endothelial cells; E_P_: Peterson's modulus; E_Pm_: modified arterial stiffness; HDL: high- density lipoprotein; LDL: low-density lipoprotein; LVD: left ventricular device; RAD: radial arterial dilatation; S: solenoid valves; SMCs: smooth muscle cells; TC: total cholesterol; TG: triglycerides.

## Competing interests

The authors declare that they have no competing interests.

## Authors' contributions

All authors participated in the design, interpretation of the studies, analysis of the data and review of the manuscript. IK, EA, and DM conducted the experiments; MK, AP, EK, and DD supplied critical reagents; IK, EA, DM wrote the manuscript; HP performed the histological analysis; MM performed the biochemical assays; and IK conducted the statistical analysis. All individuals who made contributions to this study are included as authors. All authors read and approved the final manuscript.
